# Integrating trunk endurance, dynamic stability, and in-game performance analysis in youth elite basketball players

**DOI:** 10.1186/s13102-025-01285-1

**Published:** 2025-09-24

**Authors:** Anna Gál-Pottyondy, Zsófia Pálya, Lukasz Trzaskoma, Rita M. Kiss

**Affiliations:** 1 Doctoral School of Sport Sciences, Hungarian University of Sport Science, Alkotás street 42-4, 1525 Pf. 69, Budapest, H-1123 Hungary; 2 The Hungarian Basketball Federation, Budapest, Istvánmezei Út 1-3, Budapest, H-1146 Hungary; 3 Department of Mechatronics, Optics and Engineering Informatics, Faculty of Mechanical Engineering, Budapest University of Technology and Economics, Műegyetem rkp.3, Budapest, H-1111 Hungary; 4 Department of Theory of Sport, Faculty of Physical Education, Józef Piłsudski University of Physical Education in Warsaw, Marymoncka 34, Warsaw, 00-968 Poland

**Keywords:** Trunk muscle endurance, Dynamic balance, Basketball, Match monitoring

## Abstract

**Background:**

Physical preparation in basketball is essential but often limited by training time and prior focus on tactical and technical skills. While postural stability is known to assist injury prevention and performance, its specific impact on game-related performance remains unclear. With the development of motion analysis systems, the numerical values of external and internal loads measured during matches have become measurable. This study aimed to examine the relationship between postural stability and game-related performance and introduce a method for visualizing key competencies.

**Method:**

Twenty-three U16 female basketball players (age = 15.22 ± 0.82 years, mass = 66.3 ± 8.85 kg, high = 174.0 ± 8.2 cm) participated from the Hungarian first league. Postural stability was assessed via plank test and one minute dynamic standing balance tests, while game-related performance was monitored through match-derived internal and external load values measured by WIMU PRO™ System, alongside statistical data derived from the official box scores. After the postural stability measurements, we monitored an official basketball match, which was conducted according to official International Basketball Association (FIBA) rules. For clear and comprehensive presentation, we combined the game-related performance indicators using Principal Component Analysis (PCA).

**Results:**

A moderate correlation (0.5 < *r* < 0.8, *p* < 0.05) was found between the game-related performance and postural stability variables. However, plank test indicators showed no significant correlations with game-related performance variables, except for bad throws (r = 0.56, *p* = 0.037), the postural error (*PE*), a variable reflecting compensatory movement during fatigue in the plank test, correlated with balance indicators (r = 0.63, *p* = 0.014). Mediolateral balance control correlated with explosive game-related performace metrics, including maximum acceleration (*r* = -0.65, *p* = 0.01), deceleration (*r* = 0.56, *p* = 0.035), and steals (*r* = -0.52, *p* = 0.05). PCA proved effective in creating game competency scores, enabling a graphical representation of game-related performance.

**Conclusion:**

Findings suggest that trunk endurance alone was not directly related to game-related performance, while dynamic balance metrics showed moderate correlations. The PE values provide deeper insights into the balance-trunk stability relation. The dynamic balance test could support player monitoring, and the PCA based method facilitates player profiling.

## Background

Although basketball players' physical preparation is crucial in modern, fast-paced basketball, time is often limited due to competition schedules and focus on technical/tactical aspects. To optimize fitness preparation, it's essential to understand which physical abilities impact game-related performance *(GP*), which is characterized by game-related physical values *(GRP)* and game-related statistical values *(GRS).*

Zemkové et al. [1] highlight the role of postural stability in sport-specific movements, with trunk muscles playing a key role in injury prevention and performance [2–4]. Most studies on trunk muscle strength and stability focus on training methods and separately for individual basketball-specific movements and abilities such as throwing [1, 5–7], jumping [8, 9], and running performance [10] and agility [9] and balance ability [1, 3, 11, 12]. Although trunk muscle development has a positive effect on other physical performances, except for running performance, there is no debate that trunk muscles play a significant role in the case of ball games, including basketball [1, 6, 13], it is not clear what impact it has on *GP* [1, 9, 11].

Although assessing postural stability is essential, existing training and testing protocols are often insufficient [14]. Luo et al. [9] suggest incorporating balance training to improve stability, with functional training enhancing balance, which aligns with the fact that most studies have emphasized the use of various balance tests alongside trunk strength assessments [1, 3, 11–13]. Halabchi et al. [13] suggest that both static and dynamic postural control should be measured, with static balance tests potentially revealing postural deficits in elite basketball players. While dynamic balance is well-developed in basketball players, static balance may be weaker due to less frequent use. Standard tests like the Star Excursion Balance test and Y-balance test help identify injury risk and differentiate playing positions [15–18] but they don't assess adaptive responses to external disturbances. Given the aforementioned dynamic nature of basketball, players are often exposed to various collisions and abrupt changes in direction, and the ability to adapt to such challenges is critical for advanced dynamic balance control.

However, the literature is inconsistent regarding which trunk muscles are key for maintaining the plank posture [19, 20] and the plank test is not ideal for assessing trunk muscles in isolation for healthy athletes [21]; it is commonly used or recommended in studies on trunk muscles [9, 22]. The plank test is simple to perform and measures static trunk strength endurance in field conditions [4] and can be objectively evaluated with motion capture [19, 23].

Although trunk muscles and balance ability are well-studied, there is no consensus on their effect on *GP*, and research on their role in elite and female athletes is limited [1, 11]. Therefore, the first goal of the present study was to examine the relationship between trunk muscle endurance – assessed vis prone plank test – dynamic balancing ability – characterized by the result of unidirectional balance board measurement -and *GP* using match-related *GRS* and *GRP* data*.* It was firstly hypnotized that postural stability, as measured by plank and dynamic balance test, would be associated with *GP* in U16 female basketball players*.*

Physical assessments of basketball players often involve multiple tests, but presenting these results to coaches is typically complex and unclear [24]. An example of this can be found in previous studies, where, alongside the use of numerical performance indicators, visual tools have also been introduced to support decision-making systems that assess athletic performance [25–27]. According to Gomez-Carmona [28], Principal Component Analysis (PCA)*,* a dimensional reduction technique, can be applied based on multiple physical assessments to create a composite physical index suitable for the combined evaluation of the basketball player. Our second goal was to develop a straightforward, comprehensive method for presenting multi-variable data, making it understandable for coaches and medical staff. Secondly, we hypothesizes that PCA can effectively visualize and interpret *GRP* indicators from matches and the results of the physical tests, and the ability indices of match performance could be simultaneously presented using a web chart.

## Methods

### Experimental approach to the problem

The authors used a cross-sectional study to assess if there is an association between postural stability and *GS* in U16 female basketball players. The present study design involved two postural stability measurements and the game monitoring of a basketball match. Stability assessments included a prone plank test to evaluate trunk muscle endurance, followed by a 1-min-long dynamic balance test on an omnidirectional balance board. The plank test is an objectively measurable and evaluable method [19, 23] for assessing trunk muscle endurance [4] while measuring standing balance with the aforementioned dynamic nature could serve as an effective method for evaluating the dynamic balancing ability of basketball players. Player performance was monitored in the match closest to the measurement date. Gameplay was conducted according to official International Basketball Association (FIBA) rules.

### Subjects

Twenty-three U16 (under-16) level female basketball players (age = 15,22 ± 0,82 years, mass = 66,3 ± 8,85 kg, high = 174,0 ± 8,2 cm) of two teams of Hungarian first league participated in the present study. The players had a valid sports medical certificate. The inclusion criteria specified that players must be healthy, have successfully completed and passed two physical tests, have played at least ten minutes in the monitored basketball game, and the step balance, which represents the percentage of decompensation between the intensity of steps taken with the right and left foot, should not exceed 5%. Based on the inclusion criteria, fourteen players (seven to seven from the two basketball teams) were included in the final statistical analysis of the present study. All players, coaches, and parents were informed both verbally and in writing about the measurement protocol, requisites, benefits, and risks. They agreed to participate by providing their written consent prior to the start of the experiment, which was conducted in accordance with the Declaration of Helsinki. The present study also received institutional approval from Ethics Committee Hungarian University of Sport Science (TE-KEB/17/2021).

### Experimental procedures

Players performed a prone plank test following previously validated protocols [23]. Once the correct plank posture was achieved, participants were instructed to hold the position as long as possible without receiving further corrections, even if their posture deviated. The shape of the spinal curve was recorded using a marker based OptiTrack (NaturalPoint Inc.; Corvallis, Oregon, USA) optical motion capture system at a sampling frequency of 120 Hz. An objective cutting point can be identified using the lumbar lordosis (LL) and thoracic kyphosis (TK). spinal curvatures [29], allowing for the determination of an objective test duration (*T*_*cut*_), and the postural error (*PE*), alongside the original test duration (*T*_*measured*_). *PE* was calculated based on the areas under the time curves of LL and TK [19, 23].

After completing the plank test, participants performed a 1-min balance task on an omnidirectional balance board (DOMYOS, DECATHLON; Villeneuve-d’Ascq, France) equipped with pressure sensors. Foot pressure data were collected using an F-scan in-shoe pressure measuring system (Tekscan Inc.; Boston, Massachusetts, USA), with the data collection unit secured at the participants’ waist. The pressure soles were calibrated for each individual in advance. Players, assisted by an examiner, were asked to stand on the in-soles placed on the balance board. Once the pressure sensors were connected to the measuring unit, and the participant assumed an upright standing position, external support was terminated, and the 1-min dynamic balance test began. During the test, participants were instructed to maintain their balance as steadily as possible without any prior instructions. At the end of the measurement, participants were assisted in leaving and dismounting the device.

The recorded pressure data were exported as an uncompressed.AVI file from the F-scan clinical v7.55 measurement manager software at a sampling frequency of 40 fps. The pressure values for each load cell can be determined using the scaling obtained from the pressure sole calibration and the color values of the exported videos. Based on these pressure values and the physical dimensions of the cells, the medio-lateral *(ML)* and anterior–posterior (AP) positions of the center of pressure (CoP) were calculated for each frame. Postural stability was quantified using traditional parameters, including the total path length (*Path*) and the area of the 95% confidence ellipse (*A*_*95%*_). According to Nagymate et al., the *Path* can be determined as the length of the total CoP trajectory, while *A*_*95%*_ is the smallest ellipse encompassing 95% of the CoP points, calculated using the eigenvalues of the covariance matrix [29]. To provide a more detailed analysis of stability, the complexity and the regularity of the CoP movement were characterized using sample entropy (*SampEn*) and fractal dimension (*FD*). The mediolateral (x) and anterioposterior (y) coordinates were used for these calculations. *SampEn* was determined using the PhysioNet toolkit with parameters *m* = 2 and *n* = 0.2, as recommended in the literature (for our sample size of *N* = 2400) [30–32]. *FD* was computed using Higuch’s method [33], with the tuning factor *k*_*max*_ interpolated for each trial. The optimal values were *k*_*max,X*_ = 50 for ML direction and *k*_*max,Y*_ = 30 for AP direction.

The *GRP* of basketball players was monitored using WIMU PRO™ Real Track System SL. (Almeria, Spain) The RealTrack System collected data only while players were actively competing, including during free throws. Each player wore the same microtechnology device (81 × 45 × 15 mm, 70 g) [34, 35] positioned in the centre of their upper back within an adjustable top. In previous studies WIMU PRO™ ^was^ used to measure and monitor basketball players during matches [36, 37], demonstrating good accuracy (for the x-position of 5.2 ± 3.1 cm and for the y-position of 5.8 ± 2.3 cm) and inter- and intra-unit reliability for ultra-wideband positioning (for the x-coordinate ICC = 0.65 and for the y-coordinate ICC = 0.85) [34, 38]. Data on external demands were downloaded and analysed using SPRO™ software (version 977, RealTrack Systems).

Building on methodologies from previous research, several external load metrics were defined [39, 40], playing time *(PT)* total distance covered with acceleration above 1,12 m · s − 2 per one minute (*ExplDis/min*), Distance covered above the absolute sprint speed threshold (default threshold set at 24 km/h)* (SprintDis/min)* average speed (*Speed*_*AVG*_), maximal speed (*Speed*_*MAX*_), maximal acceleration (*Acc*_*MAX*_), maximal deceleration (*Dec*_*MAX*_), number of high intensity (> 3 m · s − 2) accelerations per one minute (*HIAcc*_*N*_*/min*), number of high intensity (< −3 m · s − 2) decelerations per one minute (*HIDec*_*N*_*/min*), the distance of high intensity accelerations per one minute (*HIAcc*_*D*_*/min*) and distance of high intensity decelerations per one minute (*HIDec*_*D*_*/min*).

Following *GRS* variables were derived from the official box scores and to ensure comparability, these values were normalized by playing time: points scored per one minute *(PTS/min*), field goal per one minute *(FG/min)* the percentage of successful and missed shots *(%)*, rebounds per min *(REB/min),* steals per one minute *(STL/min)* assists pr one minute *(AS/min)*, foul dawn per one minute *(FD/min)*, turnovers per one minute *(TO/min),* and bad throw per one minute *(BT/min)*. Player performance was evaluated using the *VAL* index, a comprehensive performance metric commonly utilized by the Hungarian Basketball Federation. Similar to the *Efficiency (EFF*) index, one of the most widely applied indicators for comparing overall player performance, the *VAL* index is calculated from various *GRS* variables. However, unlikely *EFF*, the *VAL* index also includes drawn fouls, providing a more detailed assessment of player contributions (*VAL* = *PTS* + *REB* + *STL* + *AS* + *FD* + *BS*-*TO*-*BT*). To ensure comparability, *VAL* was normalized by playing time too *(VAL/min).*

### Statistical analyses

Prior to the statistical analysis, the calculated variables underwent a normality test using the Jarque–Bera test with a significance level of 0.05. Among the tests used to examine the normal distribution, this is one of the most robust and particularly effective for smaller sample sizes [41]. 93% of the calculated 30 variables passed the normality test. Consequently, the classical Pearson correlation was applied to assess the potential relationship between the postural stability parameters (*T*_*cut*_*, T*_*measured*_*, PE, Path, A*_*95%,*_* SampEn*_*ML*_*, SampEn*_*AP*_*, FD*_*ML*_*, FD*_*AP*_) and the *GP* parameters. The significance level was set to $\alpha =$ 0.05. According to Chan the following guideline was applied on the strength of the linear relationship: *r* > 0.8 meant strong correlation, 0.8 > *r* > 0.5 meant moderate correlation, *r* < 0.5 resulted fair or weak correlation [42]. Given the exploratory nature of this study, multiple comparison corrections such as Bonferroni were not applied. While such methods reduce the risk of Type I errors, they may increase the likelihood of Type II errors, potentially obscuring meaningful findings.

PCA identifies common signal structures or modes in multi-dimensional time series, producing principal components (PCs) that represent the weighted sum of original variables [43]. The first PC, associated with the largest eigenvalue, explains the largest variance of the entire dataset; thus, it can be considered the most important PC. Subsequent PCs are ranked by decreasing importance. One of the primary objectives of the present study was to characterize athlete performance clearly and understandably based on the measured and calculated variables. Achieving this clarity required a compact and concise representation of player sports competencies. Simultaneously, data reduction is needed to preserve the integrity of the original dataset, ensuring no significant loss of information. This challenge was particularly pronounced for the *GRP* variables recorded during matches, as the WIMU system records extensive data describing various aspects of gameplay. To address this, the defined variables were categorized into three subgroups based on their relevance to specific sport competencies: anaerobe endurance (*ExplDis/min, SprintDis/min, Speed*_*AVG*_), explosiveness (*Speed*_*MAX,*_* Acc*_*MAX,*_* Dec*_*MAX*_), and agility (*HIAcc*_*N*_*/min, HIAcc*_*D*_*/min, HIDec*_*N*_*/min, HIDec*_*D*_*/min*). PCA was independently performed on these subgroups after standardization. The weights of the first (most dominant) PCs were used to describe the competencies and served as the basis for constructing the game competency scores.

All the statistical analyses were performed using Matlab's *Statistics and Machine Learning Toolbox* (version R2022b, The MathWorks Inc.; Natick, Massachusetts, USA).

## Results

**Fig. 1:**
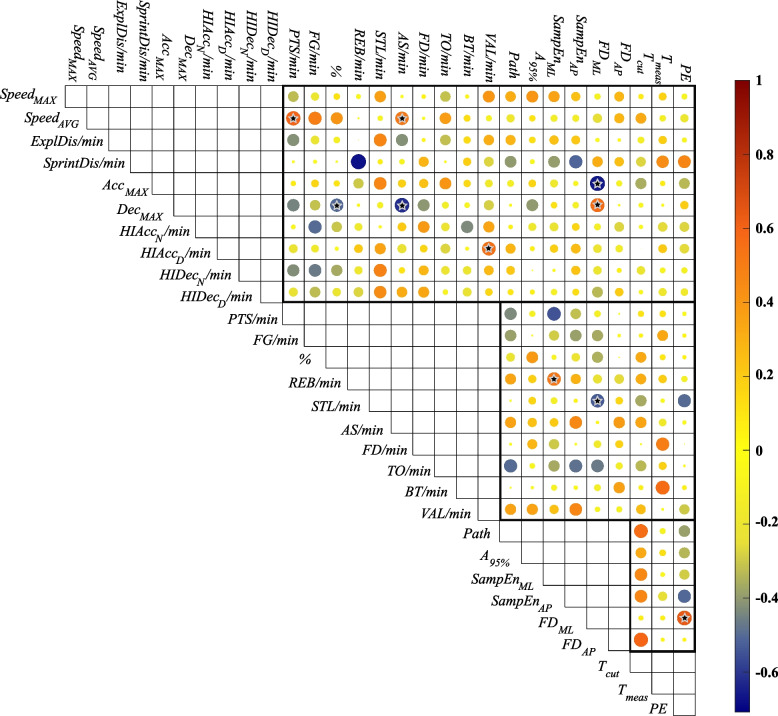
Correlation plot, stars indicate the significant (*p* < 0.05) correlations with positive meaning

The supplementary document contains all the values measured and calculated during the present research. Considering the interactions between the different variable groups (*GRS, GRP,* plank test, balance test, and ability indexes), only moderate correlations were found between variables from distinct stability tests and *GP* variables *(*Fig. 1*).*

Significant (*p* ≤ 0.05) moderate and positive correlations were found between *Speed*_*AVG*_ and *PTS/min* (*r* = 0.59, *p* = 0.024), *Speed*_*AVG*_* and AS/min* (*r* = 0.52, *p* = 0.05), *Dec*_*MAX*_* and FD*_*ML*_ (*r* = 0.56, *p* = 0.035), *HIAcc*_*D*_*/min and VAL/min* (*r* = 0.54, *p* = 0.041), *REB/min* and *SampEn*_*ML*_ (*r* = 0.53, *p* = 0.05), *BT/min and T*_*measured*_ (*r* = 0.56, *p* = 0.037), *FD*_*ML*_ and *PE* (*r* = 0.63, *p* = 0.014), *FD*_*AP*_ and *T*_*cut*_ (*r* = 0.58, *p* = 0.027) and *Path* and *T*_*cut*_ (*r* = 0.55, *p* = 0.04). Significant (*p* ≤ 0.05) moderate and negative correlations were found between *Acc*_*MAX*_ and *FD*_*ML*_ (*r* = −0.65, *p* = 0.01), and *AS/min* (*r* = −0.61, *p* = 0.018), *STL/min* and *FD*_*ML*_, (*r* = −0.52, *p* = 0.05).

When interpreting the moderate and strong correlations, it is important to consider the practical meaning of the variables. For among the parameters under consideration, some represent improved performance with higher value, such as most *GRP* and *GRS* variables, except *Decc*_*Max*_, *TO/min* and *BT/min* as well *as SampEn, T*_*cut*_ and *T*_*measured*_. In contrast, for others, better performance is indicated by lower values, such as *Path* length and *A*_*95%*_, as well as *FD* and *PE*.

The coefficient for PC1 in each game competency score and its explained variance are presented in Table 1. The aim of PCA was not to reduce the dimension of the entire GRP dataset, but rather to extract and interpret a single principal component within specific variable subsets. The associated component scores indicate the relative importance of each variable within the identified principal component with the highest relevance. Let us consider the subset related to anaerobe endurance. The explosive distance (*ExplDis/min*) indicator emerges as the most influential variable in PC1. The given WIMU measurement system typically registers sprint distance (*SprintDis/min*) values as zero for female athletes; however, exceptionally fast players occasionally exhibit non-zero values. Thus, when this value is non-zero, it contributes positively to the anaerobic endurance score. In contrast, *Speed*_*AVG*_ appears with a negative coefficient in PC1. This does not imply that higher average speed universally reduces anaerobic endurance. Rather, within this dataset and for that PC1 with the largest variance, *Speed*_*AVG*_ is negatively weighted to better differentiate between the participants. In higher-order components with smaller covered variance, this pattern reverses, and *ExplDis/min* receives a negative coefficient, while *Speed*_*AVG*_ contributes positively.

**Table 1:** The competency scores derived from the PCA analysis

name	anaerobe endurance		explosiveness		agility	
Var1	*ExplDis/min*	*0.701*	*Speed*_*MAX*_	*0.116*	*HIAcc*_*N*_*/min*	0.468
Var2	*SprintDis/min*	*0.207*	*Acc*_*MAX*_	*0.719*	*HIAcc*_*D*_*/min*	0.415
Var3	*Speed*_*AVG*_	*−0.682*	*Dec*_*MAX*_	*−0.685*	*HIDec*_*N*_*/min*	0.568
Var4					*HIDec*_*D*_*/min*	0.534
Explained variance [%]		58.0		51.3		70.3

Two main bases were established for constructing the graphical representation of the measured and calculated variables. Conversely, *GP* was assessed using game competency scores derived through *PCA* from the *GRP* variables, combined with the *VAL/min* index. On the other hand, based on the correlation analysis, it was evident that the indicator representing the execution time of the plank test is not a relevant factor for balance and match-specific performance. Considering the result of the correlation analysis, the *PE* variable was used to present the results of the plank test. For the dynamic balance test, traditional measures quantify the magnitude of the resultant CoP throughout the trial. In contrast, nonlinear measures (entropy and fractal dimension) assess CoP dynamics separately in each direction. Thus, a comprehensive evaluation of the dynamic balancing ability requires considering both traditional and nonlinear measures. Regarding the nonlinear balance parameters, PCA-based data reduction was also considered; however, due to the separate representation in the ML and AP directions, along with the different mathematical behavior (larger *SampEn* and smaller *FD* belonging to the advanced balancing strategies), the result variable would have been overly complex and less meaningful to express the equilibrium ability. In our case, correlation results indicated that *Path* had a more significant influence on other variables among the traditional measures, making it an optimal choice for characterizing the overall CoP displacement. Consequently, the following six indicators assessed balance and core stability: *PE, Path*, *SampEn*_*ML*_*, SampEn*_*AP*_*, FD*_*ML*_*, and FD*_*AP.*_

**Fig. 2:**
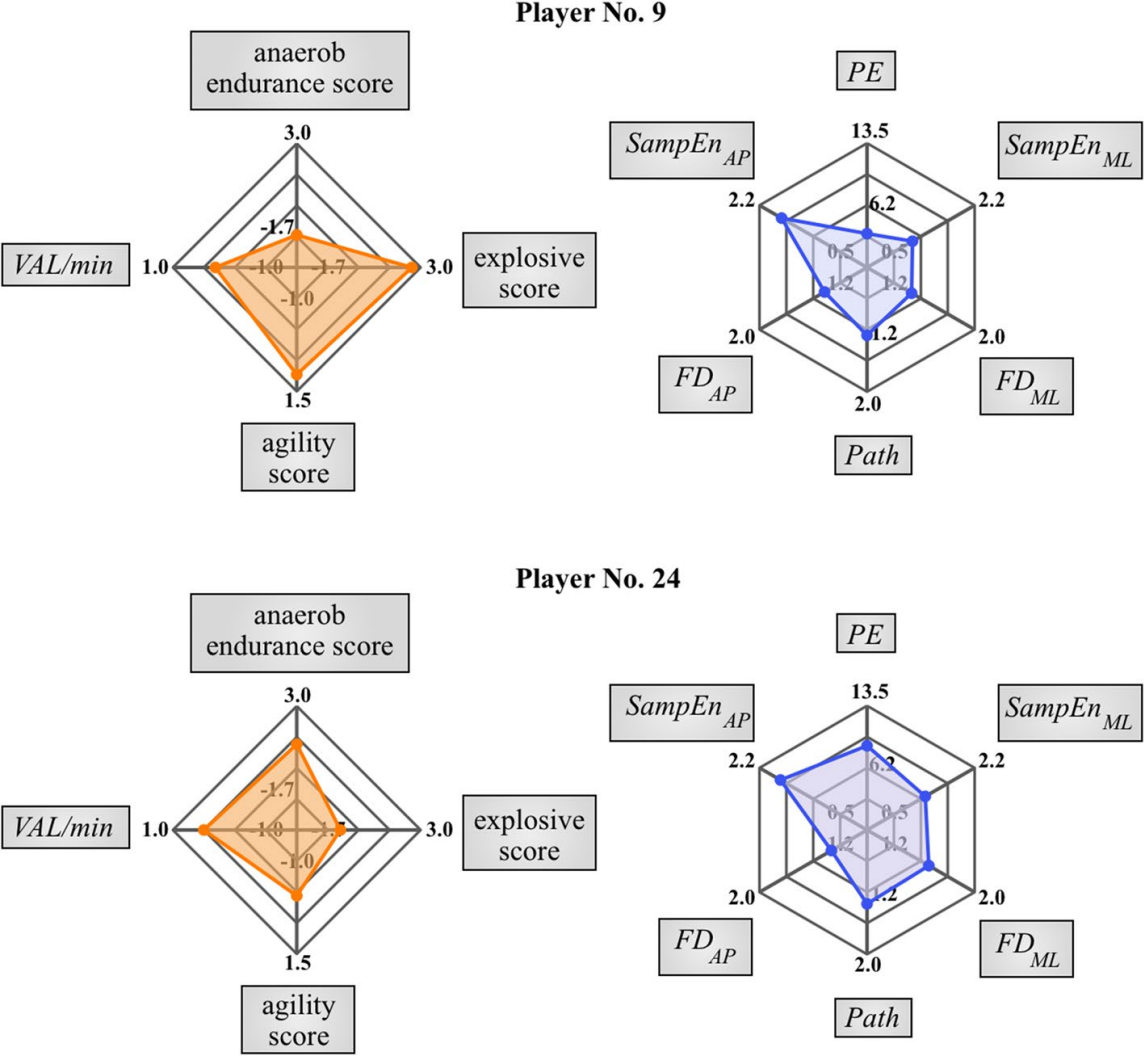
Spider plot containing the game competency scores and stability variables of player No. 9 and Player No. 24

The two collections of competencies are shown in a separate web chart to profile the player’s game performance and balancing ability. These graphical results must be evaluated individually, not as comprehensive team statistics; therefore, it also allows for identifying specific areas where each player could improve their performance. For example, Fig. 2 shows that player No. 9 is an explosive, agile player with relatively high balance and plank values, and the area to be developed in her case is anaerobic endurance. Although No. 24 demonstrates high-level anaerobic endurance and basketball-specific efficiency, her performance in other areas remains relatively low.

## Discussion

Our primary goal was to examine whether postural stability assessed by plank test and 1-min-long dynamic balance test has a measurable effect on *GP*. It was first hypnotized that there would be a correlation between the postural stability of U16 female basketball players characterized by the plank and dynamic balance tests and *GP* characterized by *GRP* and *GRS.* We found significant moderate-strength correlations between the balance test and *GP* indicators, mixed with positive and negative associations. The correlations with a positive meaning for us were as follows: *Dec*_*MAX*_* and FD*_*ML*_ (*r* = 0.56, *p* = 0.035, CI_95%_ [−0.84, −0.04]), the *REB/min* and *SampEn*_*ML*_ (*r* = 0.53, *p* = 0.05, CI_95%_[0.00, 0.83]), *Acc*_*MAX*_ and *FD*_*ML*_ (*r* = −0.65, *p* = 0.01, CI_95%_[−0.88, −0.19]), *STL/min and FD*_*ML*_ (*r* = −0.52, *p* = 0.05, CI_95%_[−0.83, 0.00]). The values of the plank test did not correlate with *GRP,* and among *GRS,* only the *T*_*measured*_ correlated with *BT/min*, which holds a negative meaning.

To the best of our knowledge, no previous research has compared *GP* with balancing ability. While the connection between balance ability and sports injury risk is well-documented [44, 45], its relationship with athletic performance remains unclear. Notably, our findings highlight moderate correlations between *Acc*_*MAX*_ (*r* = −0.65, *p* = 0.01, CI_95%_[−0.88, −0.19])*, Dec*_*MAX*_ (*r* = 0.56, *p* = 0.035, CI_95%_ [−0.84, −0.04]), and *STL/min* (*r* = −0.52, *p* = 0.05, CI_95%_[−0.83, 0.00]) with *FD*_*ML*_. Higher maximum acceleration and deceleration values indicate more explosive movements requiring substantial muscle work and peak power output. Similarly, elevated *STL/min* scores reflect increased coordination demands and rapid muscular exertion during ball-stealing movement. These findings imply that explosive movements may require a higher degree of neuromuscular coordination. Considering the practical interpretation of the *FD* value, an *FD* equal to 1 represents smooth, controlled movements, while an *FD* equal to 2 indicates fully chaotic behavior [44]. These correlations may suggest that athletes with greater *Acc*_*MAX*_*, Dec*_*MAX*_, and *STL/min*, achieved through higher force exertion, tend to have lower FD values, implying more deliberate and controlled balance coordination. Only the ML direction showed correlations with *Acc*_*MAX*_*, Dec*_*MAX*_, and *STL/min* among the two directional *FD* values. This aligns with the understanding that ML balance adjustments, which involve shifting the center of mass from side to side, are more energy-consuming than the primarily ankle-driven AP balance control [45]. A higher entropy value reflects a more structured and controlled balance coordination, indicating a player's enhanced ability to adapt to unexpected external disturbances [44]. Given that ML balancing is more demanding, it is expected that *GP* parameters requiring maximal muscular effort would be linked to this direction of balance compensation. The observed moderate correlation between *SampEn*_*ML*_ and *REB/min* (*r* = 0.53, *p* = 0.05, CI_95%_[0.00, 0.83]) may indicate that athletes with more advanced balance control, potentially reflecting greater adaptability to rapid postural adjustments, tend to be more effective in rebound situations. This finding highlights a potential for improving defensive strategies by emphasizing balance training to enhance a player's ability to react dynamically in rebound situations. Based on these findings, we accept our first hypothesis since the balance test and plank test indicators are correlated with the *GP;* however, in the case of the plank test, it was a negative meaning relation. The lack of significant associations between plank test outcomes and *GP* values warrants further consideration and should be regarded as an important finding.

Although experts have found correlations between the plank test and other physical ability tests [46, 47] and even specifically recommend for basketball players muscle strength development through plank holds [9], based on our results, it seems that trunk muscle endurance is not the primary factors influencing basketball-specific performance. It is in line with Cengizhan et al. [48] who didn’t find a significant relationship between trunk stability and agility among basketball players, and with Sharrock et al. [49] who found only weak correlations between medicine ball throw and trunk muscle endurance (*p* = 0.026, *r* = −0.322) and didn’t find correlations between results of agility, jump, and running tests and trunk stability. This result does not mean that basketball players do not need trunk muscle stability and endurance, as they are crucial for injury prevention [50] and are linked in the literature to improved physical performance in general ( 1, 5–8, 51). In line with previous research and our findings, a certain level of core muscle endurance appears to be necessary for basketball performance; however, improvements beyond this threshold may not yield additional sport-specific benefits. In the present study, we examined female basketball players competing in the U16 first division whose trunk strength endurance was at an adequate level. Accordingly, plank test performance beyond this minimal threshold did not appear to be associated with further improvements in *GP* values. This aligns with the observation that, for trained athletes, a minimum of a 6-week trunk strength and balance development program is required to yield improvements in other performance indicators [52]. Another study highlights that the development of trunk strength exercises will only be effective for a particular movement if they are appropriately functional and sport-specific [11].

The literature contains numerous studies that explore the relationship between trunk stability and balance ability, and based on the time measurement, previous research has found no correlation between trunk muscle endurance and dynamic balance ability [53, 54]. Similarly, the present study found correlations with negative significance between the plank test durations and the balance test: *FD*_*AP*_ and *T*_*cut*_ (*r* = 0.58, *p* = 0.027, CI_95%_[0.08, 0.85]) and *Path* and *T*_*cut*_ (*r* = 0.55, *p* = 0.04, CI_95%_[0.03, 0.84]). For *PE*, we found correlations with positive meanings between the two tests, specifically between the *FD*_*ML*_ and *PE* variables (*r* = 0.63, *p* = 0.014, CI_95%_[0.16, 0.87]). The *PE* value reflects how an individual performs the plank test [23]. A low *PE* value occurs when the person actively corrects their posture after small displacements, returning to the starting position. In contrast, a high *PE* value is associated with situations where the individual does not correct their posture during the plank, gradually deviating from the initial position [23]. In the present study, we introduced the use of *PE* values to quantify the relationship between a dynamic balance test, which requires active postural regulation, and the plank test, which assesses trunk muscle stability and endurance. This approach assumes that balancing ability is more closely associated with the fine micromovements necessary to maintain posture than with the duration for which a static position can be held. This novel methodology provides valuable insight into the functional link between trunk stability and balance control.

Our second goal was to analyze the measured variable groups and game competency scores through a practical and applicable method. In line with Gómez-Carmona et al. [28], the PCA method can be applied to provide a combined characterization of physical assessments and performance indicators measured during matches. Therefore, alongside some necessary variable reduction, it was aimed to propose a graphical solution for visualizing *GP* and postural stability. The web charts developed for this purpose, which display game competency scores, the *VAL/min* index as a summary of *GRS* indicators, and the most essential postural stability parameters, are suitable for analyzing the multivariable player profiles clearly and understandably for coaches and medical staff to analyze player performance.

**Fig. 3:**
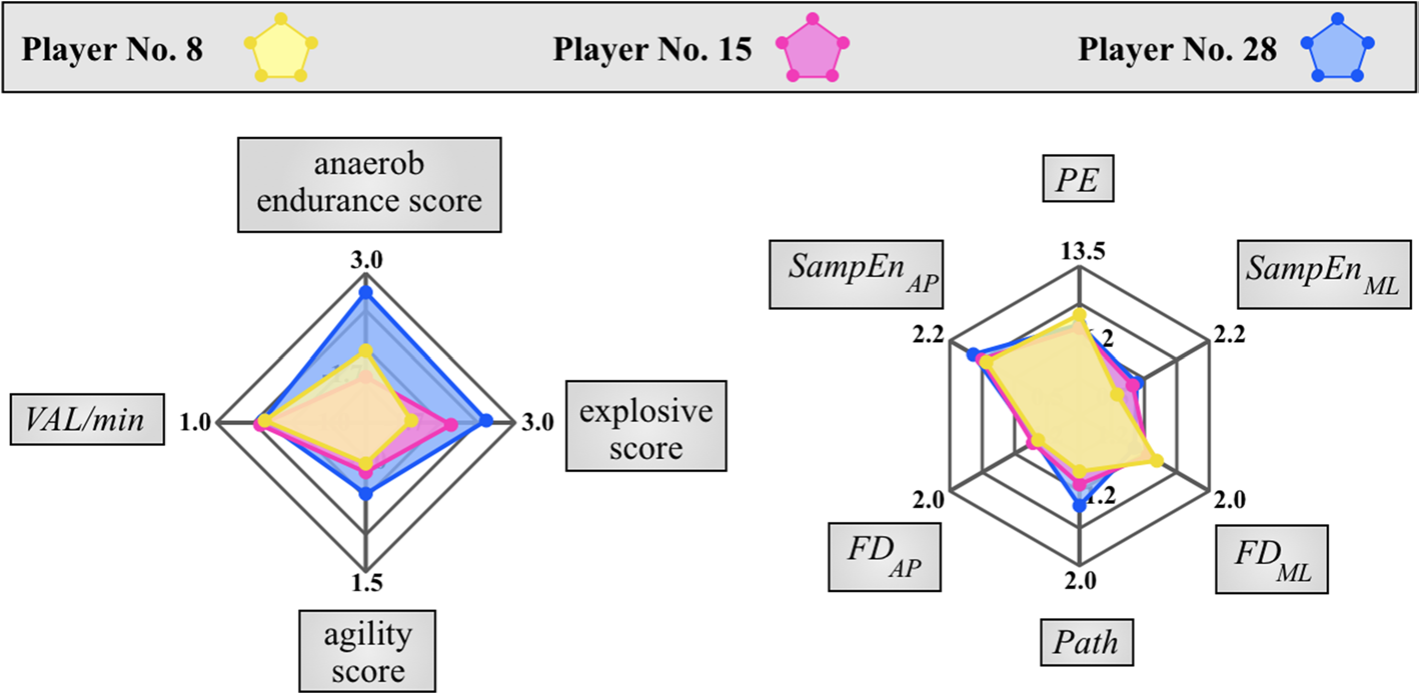
Example for comparing players’ competencies

By looking at the web charts, it is easy to identify the strengths and weaknesses of individual players. Players No. 8, No. 15, and No. 28 have similar *VAL/min* index and explosiveness score, but it is clearly visible that player No. 8 excels in agility and balance, player No. 28 stands out for anaerobic endurance while Player No. 15 has average values in both anaerobe endurance, agility, explosiveness, balance and trunk stability (Fig. 3). By representing the ability scores on a web chart, the analyst coach has the opportunity to present the players' performance in a clear and easily understandable way.

One of the primary limitations of this study is the relatively small sample size, which may limit the generalizability of the findings and the statistical power of correlation analyses. Although effect sizes were reported to aid interpretation, the results should be interpreted with appropriate consideration. In the case of PCA, the ratio of observations to variables did not meet the conventional threshold for large-scale multivariate analysis; therefore, the analysis was limited to smaller, conceptually grouped subsets of variables. This approach was intended for exploratory visualization rather than drawing general conclusions based on the competency scores.

## Conclusion

The present study introduced the application of *PE* values to better quantify the relationship between dynamic balance and trunk stability, thereby underscoring the intrinsic connection between these two components of athletic performance. The *PE* value serves as an effective representation of trunk muscle stability and provides a valuable complement to traditional dynamic balance assessments. Using the plank position into training protocols may offer utility not only as a core stability exercise but also as a fundamental component of conditioning evaluations across various athletic fields. Nevertheless, for sport-specific testing in basketball, our findings suggest that the plank test is useful only when *PE* values are available, as these were the only metrics that correlated meaningfully with balance indicators. Traditional plank test scores had minimal or negative associations with GP metrics.

Our findings also revealed weak to moderate correlations with positive meaning between the balance indicators and the *GP*. Players demonstrating more advanced balance control appear to engage in more deliberate coordination strategies, particularly in the energy-demanding ML direction, which is correlated with enhanced performance on measures of explosive agility. Additionally, higher entropy values suggest improved adaptability to external disturbances, which may enhance a player's ability to secure rebounds, offering a potential strategy for defensive training. A simplified implementation of the dynamic balance test (e.g., recording single-leg stance or balance tasks on a BOSU ball using markerless video analysis or affordable inertial sensor) could become a routine part of monitoring balance development during training.

When analyzing *GP*, it is helpful to categorize abilities and create competency scores to provide a comprehensive overview of a player's skills. Representing the competency scores in a web chart helps clearly present their strengths and weaknesses. Furthermore, it allows us to track a player's performance progression over the competitive season.

## Data Availability

The authors confirm that the data supporting the findings of this study are available within the article, and further detailed descriptive datasets used and/or analysed during the current study are available from the corresponding author on reasonable request.

## Abbreviations

A_95%_: Area of the 95% confidence ellipse during balance test
Acc_MAX_: Maximal acceleration
AP: Anterior posterior
CoP: Center of pressure
Dec_MAX_: Maximal deceleration
ExplDis/min: Explosive distance per one minute
FD: Fractal dimension
FIBA: International Basketball Association
GP: Game-related performance
GRP: Game-related physical values
GRS: Game-related statistical values
HIAcc_N_/min: Number of high intensity accelerations per one minute
HIDec_N_/min: Number of high intensity decelerations pre one minute
LL: Lumbar Lordosis
ML: Mediolateral
Path: Total path length of CoP during balance test
PCA: Principal Component Analysis
PCs: Principal component
PE: Postural error
PT: Playing time
SampEn: Sample entropy
Speed_AVG_: Average speed
Speed_MAX_: Maximal speed
SprintDis/min: Sprint distance per one minute
T_cut_: The cut-off time result of the plank test
TK: Thoracic kyphosis
T_measured_: The total, measured time of the plank test
